# Medical Clinic for the Session of 1860

**Published:** 1860-10

**Authors:** J. G. Westmoreland


### ARTICLE III.

*Medical Clinic, for the Session of* 1860. By J. G. WESTMORELAND, M. D. (Continued from page 13.)

May 11.—Case 2.—A negro child, aged 8 months; affected with diarrhea from teething. Diarrhea, properly speaking, is not the name of a disease, but the term by which one of the symptoms of enteric diseases is known.

The period of first dentition is that fraught with more danger than any other, in the life of children; particularly when it occurs during the warm season.

The danger is still farther increased by epidemic influence, tending to the production of enteric disease in whole neighborhoods, which often exits.

At this tender age the system is naturally impressible, and any source of irritation so disturbs the functions of the nervous system that irritability of the more excitable tissues is induced, which require only a slight cause to establish in them positive inflammation. The alimentary mucous surface is very susceptible to such disturbance from the irritation produced in the gum by a protruding tooth. In addition to this, various forms of functional derangement are set up by reflex nervous action or sympathy, taking their origin from these points of local irritation or inflammation. The brain itself is not unfrequently the subject of functional derangement, the symptoms of which become so prominent, that they are frequently mistaken for those of menengitis, effusion, &c.

The indications in the treatment of such cases are: firstly, to remove the primary source of irritation, by a free incision of the gum; secondly, to allay the enteric irritation, which is more readily met by such means as will quiet general nervous irritability, restrain as much as possible the frequency of the evacuations, and soothe and protect the tender surface from the contact of irritating substances in the canal—these means are opiates, astringents, and demulcents.

We present again, the case of Nervous Rheumatism examined and prescribed for before you, two days ago. You will perceive that he walks without much difficulty. He reports himself almost free from pain. Quinine, in two portions of ten grains each, without opium, to be taken, one to-night and the other to-morrow morning. Opium, it is thought, interferes with the favorable action of quinine in rheumatism, and from my observation of a few cases in which the test was made, am inclined to concur in the opinion. In cases, however, of great suffering, any counteracting influence that it may have, to the action of quinine, is overbalanced by the comfort and sleep it insures.

CASE 3.—We have before us another case of the same affection. The boy is some twenty years old, and has been for several years, subject to paroxysms of intense suffering in various parts of the body, of a neuralgic character, while at other times pain of a rheumatic variety is felt in the hips, thighs and other parts. On each leg of this patient is found a chronic ulcer, of doubtful origin. Whether the deranged condition of the nervous system had anything to do with their protection or continuance, is somewhat uncertain.—Make the following prescription :

℞—Sulph. Quinine, grs. xx.

Carb. Iron, grs. xl.

Mix and make 8 powders. One to be taken night and morning.

Case 4.—The young man is troubled with partial deafness in one ear; complains of no pain ; has no discharge from the meatus externus, and does not discover the bubbling sensation and other symptoms of disease of the Eustachian-tube. In all probability, depends on an accumulation of cerumen on the membrana tympani. With this view we will inject warm solution of soap into the ear and let it remain for a few moments in order to soften the suspected wax, then inject with a pretty forcible stream, so as to dislodge and rinse it from the meatus.

Case 5.—White female, 50 years old—the subject of Tubercular Phthisis, for 6 years. In this case are found some of the physical signs of disease important to be understood in order to correct diagnosis. These signs are detected by auscultation and percussion—auscultation of the voice and respiration. Certain sounds are communicated to the ear when placed in contact with the walls of the chest, from the respiration and from the voice, when the respiratory organs are in a healthy condition. Changes in these sounds are made by disease of the organs. It is necessary, Gentlemen, that you learn these normal sounds, in order to the detection of any variation produced by disease. We have in the case before us, variation from the natural vesicular murmur and bronchial respiration; indeed, an absence of these at a particular point, and at this point we hear a sound in respiration called *cavernous ronchus.* When the patient is directed to speak while the ear is still applied, instead of the natural sound of the voice, we have that variation called *Pectoriloquy.* These are caused by the destruction of a portion of the lung, leaving a cavity. Another abnormal sound of the voice—produced by a condition of the lung very different from that by which pectoriloquy is caused—so very nearly resembles pectoriloquy that the aid of percussion is necessary to distinguish the two conditions. It is *Bronchophony*, a sound made by hepatized or solid portion of lung intervening between the point where the ear is placed and the large bronchia, particularly when the latter are dilated. When a finger of the left hand is placed upon the chest and suddenly struck by the points of the two fore-fingers of the right hand, a certain degree of resonance is produced when the lung beneath is healthy. This resonant sound varies in disease according to the changes which take place in the lung; so that when doubt exists in regard to the sound of the voice, whether it be bronchophony or pectoriloquy, the difficulty in diagnosis may be readily removed by determining through percussion whether a portion of lung be absent or solidified. In this case, you will observe, as I percuss the point where pectoriloquy is heard, not the sound which is produced by striking a solid but a hollow substance. You will discover also, Gentlemen, by that mode of examination called *inspection*, that there is a disproportion in the size and shape of the right and left sides of the chest. The right side is flattened, while the left maintains its natural form. This is not owing to the cavity in the right lung which we have detected by auscultation and percussion, but to the solid and inactive condition of other portions of the same lung, whereby its dimensions are lessened, and the parieties of the chest adapt themselves to the size of the lung, from external atmospheric pressure, while the internal through the respiratory tubes is cut off by the solid state of the lung.

The treatment consists in directing the patient to use nourishing diet, whisky, opium, and the inhalation of the fumes of Iodine.

